# Beyond the Pump: A Narrative Study Exploring Heart Memory

**DOI:** 10.7759/cureus.59385

**Published:** 2024-04-30

**Authors:** Abdulkreem Al-Juhani, Muhammad Imran, Zeyad K Aljaili, Meshal M Alzhrani, Rawan A Alsalman, Marwah Ahmed, Dana K Ali, Mutaz I Fallatah, Hamad M Yousuf, Leena M Dajani

**Affiliations:** 1 Surgery, King Abdulaziz University, Jeddah, SAU; 2 College of Medicine, Imam Abdulrahman Bin Faisal University, Dammam, SAU; 3 College of Medicine, Al-Baha University, Al-Baha, SAU; 4 College of Medicine, Arabian Gulf University, Manama, BHR; 5 College of Medicine, Batterjee Medical College, Jeddah, SAU; 6 College of Medicine, King Khalid University, Abha, SAU

**Keywords:** cardiac transplantation, personal identity, heart-brain connection, organ transplantation, cellular memory

## Abstract

The field of organ transplantation, particularly heart transplantation, has brought to light interesting phenomena challenging traditional understandings of memory, identity, and consciousness. Studies indicate that heart transplant recipients may exhibit preferences, emotions, and memories resembling those of the donors, suggesting a form of memory storage within the transplanted organ. Mechanisms proposed for this memory transfer include cellular memory, epigenetic modifications, and energetic interactions. Moreover, the heart's intricate neural network, often referred to as the "heart brain," communicates bidirectionally with the brain and other organs, supporting the concept of heart-brain connection and its role in memory and personality. Additionally, observations from hemispherectomy procedures highlight the brain's remarkable plasticity and functional preservation beyond expectations, further underscoring the complex interplay between the brain, body, and identity. However, ethical and philosophical questions regarding the implications of these findings, including the definition of death and the nature of personal identity, remain unresolved. Further interdisciplinary research is needed to unravel the intricacies of memory transfer, neuroplasticity, and organ integration, offering insights into both organ transplantation and broader aspects of neuroscience and human identity. Understanding these complexities holds promise for enhancing patient care in organ transplantation and deepens our understanding of fundamental aspects of human experience and existence.

## Introduction and background

It has been traditionally conceived that the heart functions solely as a pump responsible for receiving, transferring, and oxygenating blood. Numerous studies, particularly those conducted after heart transplantation surgeries, have observed instances where recipients' behavior has mirrored that of the donors in certain situations and circumstances [1-3].

Emotions, traditionally attributed to the brain, have been found to have origins in the heart as well, often referred to as "heart feelings." It has been suggested that these feelings can be transferred along with the heart to recipients, who may exhibit behaviors similar to those of the donor [2]. The concept of heart memory is crucial in delving into these notions, as it parallels the brain's memory functions. Just as the brain retains memories, the heart can also store memory. Moreover, the manifestation of specific signs and symptoms in particular situations, regulated by the autonomic nervous system, further contributes to these phenomena [2].

Heart transplantation studies have revealed numerous alterations in recipients' personalities, often exhibiting traits that align with the donor's. Moreover, exosomes, which are protein-based vesicles, contribute to the transfer of information between organs and body cells. This enhances our comprehension of the diverse forms of memory present within the human body.

Neuroplasticity, on the contrary, significantly influences memory. Studies have indicated that recipients of heart transplants have demonstrated the capacity to recall information presumed to be stored in the intracardiac nervous system [3,4]. Additionally, neurotransmitters play a pivotal role in this process, serving as the primary medium for information transfer between neurons.

The body of evidence supporting the existence of heart memory is growing, propelled by advancements in medicine and research technology. It has been demonstrated that various forms of memory exist on both cellular and neurological levels. Evidence supporting the theory of "multiple forms of memory" is derived from both heart transplantation studies and molecular sciences [2].

Heart memory, encompassing emotional imprints and genetic influences, poses intriguing implications in heart transplantation [2,5]. While electrocardiography reveals persistent alterations post-transplantation, emotional imprints may transfer to recipients, potentially influencing their psyche [2,5]. Genetic composition, notably RNA and DNA sequences in cardiomyocytes, may perpetuate such memory, impacting recipients' emotional experiences [2,5]. Additionally, the nervous system's role in transmitting memory warrants further investigation. Understanding these mechanisms is vital for safeguarding recipients' emotional well-being and optimizing transplant outcomes, underscoring the need for this comprehensive review in this domain.

## Review

Heart brain

Across various cultures, including Hebrew, Christian, Chinese, Hindu, and Islamic traditions, the heart is often regarded as the seat of emotions, desires, and wisdom [6]. The notion of the "heart brain," also referred to as the "little brain" or "intrinsic cardiac nervous system," was initially proposed by Dr. J. Andrew Armour in 1991. This conceptualization suggests that the heart possesses its own neural network, comprising around 40,000 neurons capable of sensing, feeling, learning, and retaining memories. The neurons found in the heart share similarities with those present in the brain [6]. Cardiac afferent neurons are situated in both the dorsal root and nodose ganglia, along with the intrinsic cardiac and intrathoracic ganglia. The intrinsic cardiac neurons can produce spontaneous activity without relying on inputs from central or other intrathoracic neurons [7]. Consequently, the heart possesses its autonomous nervous system. From this viewpoint, the heart emerges as a system characterized by complexity and self-organization. The heart maintains continuous bidirectional communication with both the brain and the entire body [7].

Heart-brain communication

The literature has evidenced various modes of communication between the heart and the brain. These include neurological pathways involving nerve impulses, biochemical signaling through hormones, biophysical transmission via pulse waves, and energetic interactions through electromagnetic fields [8]. In contrast to the brain, the heart generates approximately 40-60 times more electrical power and 5000 times more electromagnetic power. As a result, the heart possesses the capacity to harmonize and coordinate all bodily systems, leading to physiological coherence [6]. The heart is responsible for producing and releasing multiple hormones. One such hormone is atrial peptide, also known as atrial natriuretic peptide. This hormone inhibits the release of stress hormones, diminishes sympathetic outflow, and impacts motivation and behavior [9]. Additionally, it is noteworthy that the primary source of brain natriuretic peptide (BNP) is the cardiac ventricle rather than the brain itself. Furthermore, the heart plays a role in synthesizing and releasing oxytocin, commonly referred to as the "love" or "social bonding" hormone. Oxytocin is implicated in various cognitive processes such as tolerance, trust, and social bonding [9]. The vagus nerve serves as a conduit for transmitting information from the heart and other internal organs to the brain. It terminates primarily in the brainstem, specifically within the medulla and the solitary nucleus [9]. Approximately 80% of the fibers within the vagus nerve are afferent, or ascending, in nature. As mentioned earlier, this implies that the heart transmits more signals to the brain than it receives in return [8]. Interestingly, signals originating from the "heart brain" are conveyed to the cerebral cortex via afferent neurons in the spine and the vagus nerve. Before reaching the cerebral cortex, these signals are directed to various brain regions, including the medulla, hypothalamus, thalamus, and amygdala [8]. Research indicates the existence of a pathway from the dorsal vagal complex and cardiovascular afferent signals that directly project to the frontal cortex [9].

Recipients personality changes after heart transplantation

Preferences

Food preferences: Changes in food preferences have been reported post-transplant, including both alterations in preferred food types and the quantity consumed. For instance, a 29-year-old woman who received a heart transplant from a 19-year-old vegetarian expressed aversion to meat post-surgery, previously being a frequent consumer of meat-based fast food [3,10]. Similarly, a 47-year-old male recipient experienced nausea and a desire to vomit after meals following transplantation from a 14-year-old donor with irregular eating habits [3,10]. Conversely, a 48-year-old female recipient developed a newfound taste for green peppers and chicken nuggets, foods previously disliked, after receiving her transplant from a donor who favored these items, even discovering a packet of chicken nuggets with the donor after his accident [3,10].

Musical preferences: Changes in musical preferences have also been observed post-transplant. A 45-year-old recipient started enjoying loud music, which was previously not a habit. An 18-year-old girl who received a heart from a musician reported a newfound love for music post-transplant [3,10]. Similarly, a 47-year-old man who received a heart from a young African American male began appreciating classical music, a genre he previously disliked. These changes were attributed to the donors' preferences, indicating a possible influence of the transplanted heart on the recipient's tastes [3,10].

Sexual preferences: Alterations in sexual preferences post-transplant have been documented. A male recipient of a heart from a lesbian artist reported heightened sexual desire toward women, suggesting a shift in his sexual orientation [3,10]. On the contrary, a lesbian recipient of a heterosexual woman's heart found herself attracted to men post-transplant, leading to confusion about her sexual identity. These cases hint at potential changes in sexual orientation following heart transplantation, although the mechanisms behind such shifts remain unclear [3,10].

Other preferences and aversions: Beyond food, music, and sexuality, changes in preferences for art, colors, and aversions have been noted post-transplant. For instance, a landscape artist's heart recipient developed an interest in art, while a dancer's heart recipient experienced a shift in color preferences toward cooler tones [3,10]. Contrariwise, recipients have developed aversions such as a fear of water following transplantation from a drowning victim. These cases underscore the complexity of psychological and behavioral changes that may occur post-heart transplant, possibly influenced by the donor's characteristics and experiences [3,10].

Emotional Changes

Following heart transplantation, recipients report two main types of emotional changes. First, some identify specific emotions they believe originate from the donor. For instance, a nine-year-old boy described sensing sadness and fear from his three-year-old donor, who drowned tragically. His mother revealed details of the donor's tumultuous family life, suggesting a connection between the donor's experiences and the recipient's perceived emotions [3,10,11]. Second, recipients note alterations in temperament, attributing personality traits of calmness or emotional reactivity to the donor [3,10,11].

Identity Changes

Changes in personal identity are frequently observed post-heart transplantation, revealing a profound psychological impact. Recipients often develop a deep sense of connection with their donors, attributing familial relationships or even conversing with them. For instance, a 19-year-old recipient perceives her donor as a sister, engaging in heartfelt conversations and feeling her presence in her chest. Similarly, a five-year-old boy, unaware of his donor's identity, imagines him as a younger brother named Timmy, imbuing him with a personality and history. Some recipients even experience dreams or memories aligning with their donor's identity, such as a woman envisioning a young man named Tim during a dream and later discovering her donor's name as Tim Lamirande [3,10].

Memory Changes

Some heart transplant recipients report experiencing memories that seemingly belong to their donors. These memories manifest as sensory perceptions, occurring during both wakefulness and sleep. For instance, one recipient describes sudden unusual tastes accompanied by thoughts about their donor's identity and life experiences. Another recipient feels tactile sensations corresponding to the impact of the car accident that killed their donor [3,10]. Visual information is also reported, such as a recipient experiencing flashes of light and heat resembling the trauma suffered by their donor, who was shot in the face. Another recipient describes a vivid dream of reckless driving, mirroring the circumstances of their donor's fatal motorcycle accident. These accounts suggest a phenomenon where recipients inherit memories or sensations from their donors, highlighting the intricate interplay between the mind and the transplanted organ [3,10].

Possible rationale and mechanisms for heart memory

The phenomenon of personality changes following heart transplantation has captured the imagination of scientists and the public alike, presenting a fascinating intersection of medicine, psychology, and metaphysics [3,10,12,13]. While the concept of personality is influenced by the transplantation of a vital organ may seem unbelievable, numerous studies have lent credence to the idea that the recipient may exhibit traits and preferences reminiscent of their heart donor [3,10,12,13]. These observations raise profound questions about the nature of memory, identity, and consciousness.

Among the various explanations proposed to account for these observed personality changes, one intriguing hypothesis centers on the concept of cellular memory [3,14,15]. This theory posits that memories or personality traits may somehow be encoded within the cells of the transplanted organ, in this case, the heart, and subsequently transferred to the recipient. Cellular memory challenges conventional views of memory, which have long been associated solely with neuronal processes within the brain [3,14,15].

Traditionally conceived as the capacity to acquire, store, and retrieve information, memory has primarily been linked to synaptic connections and neural circuits in the brain [16,17]. However, recent advances in genetics and epigenetics have expanded our understanding of memory beyond the confines of the nervous system [16,17]. Epigenetic modifications, which involve chemical alterations to DNA and histone proteins, can influence gene expression and have been implicated in the storage and transmission of information across generations [18-22].

The existence of epigenetic memory suggests that experiences and environmental factors can leave lasting imprints on an individual's genome, shaping not only their phenotype but also potentially influencing aspects of their behavior and personality [18-22]. This form of memory, encoded within the epigenome, provides a mechanism through which the experiences of the donor could theoretically be passed on to the recipient following a heart transplant [18-22].

Furthermore, the literature has highlighted the role of non-coding RNAs, particularly those contained within extracellular vesicles such as exosomes, in mediating intercellular communication and gene regulation. These small RNA molecules, including microRNAs (miRNAs) and long non-coding RNAs (lncRNAs), have been implicated in various biological processes, including memory formation and synaptic plasticity [23-27].

Many studies have demonstrated the transfer of long-term memory between individuals by injecting RNA extracted from trained donors into naïve recipients [23-27]. This remarkable finding suggests that RNA-mediated mechanisms could contribute to transmitting memories or behavioral traits from heart donors to transplant recipients [23-27].

Moreover, the potential role of proteins, particularly prions, in-memory storage and transmission cannot be overlooked. Initially identified as infectious agents associated with neurodegenerative diseases, prions have since been implicated in normal physiological processes, including synaptic plasticity and memory formation [28-33].

The presence of prions in exosomes raises the possibility that these proteinaceous particles could serve as vehicles for transferring memory-related molecules between cells [28-33]. Indeed, exosomes have emerged as crucial mediators of intercellular communication, facilitating the exchange of proteins, nucleic acids, and lipids between cells in various physiological and pathological contexts [34,35].

In addition to cellular and molecular mechanisms, other forms of memory transfer have been proposed, including the concept of cardiac neurological memory [3]. The heart, often referred to as the "second brain," harbors its intricate network of neurons and neurotransmitters, which play a vital role in regulating cardiac function and may also be involved in the encoding and storing of memories [3].

Furthermore, the notion of energetic memory posits that changes in the heart's electromagnetic field could influence the recipient's personality and consciousness [3]. While this hypothesis remains speculative, it underscores the interconnectedness of physiological processes and the potential for subtle energy fields to influence human experience [3].

The phenomenon of personality changes following heart transplantation represents a multifaceted puzzle that challenges our conventional understanding of memory, identity, and consciousness. While the exact mechanisms underlying these observed changes remain elusive, emerging evidence from various fields suggests that memory transfer across cellular, molecular, and even energetic levels may shape the recipient's post-transplant experience.

Functional preservation beyond expectations in hemispherectomy

Hemispherectomy, a rare neurosurgical procedure, involves removing or disconnecting one cerebral hemisphere to treat severe epilepsy unresponsive to medication, as seen in Rasmussen's encephalitis or Sturge-Weber syndrome [36,37]. Despite concerns about cognitive and personality impacts, especially in children, outcomes can be surprisingly positive due to neuroplasticity. Research indicates that while some challenges like language or memory may arise post-surgery, fundamental personality traits typically remain intact [36,37]. Though changes can occur, they're often less severe than anticipated. Patients tend to maintain interests and emotional responses, though some adjustments are expected due to the procedure's neurological effects and psychological factors [36,37]. Only a limited number of case series regarding hemispherectomy in patients with Rasmussen’s encephalitis have been documented in scientific literature. The literature has demonstrated the advantageous effects of hemispherectomy on seizure management, along with less pronounced alterations in functional outcomes compared to earlier expectations [28,38-40].

The study conducted by Thome et al. comprised a cohort of 44 patients exhibiting typical characteristics of Rasmussen’s encephalitis, including an early onset of seizures (mean: six years), frequent daily seizures, and the presence of focal motor deficits [37]. Prior to undergoing surgery, all individuals in the Thome et al. study were receiving polytherapy for epilepsy management, with 50% of them having also undergone immunomodulatory treatment. This underscores nonsurgical interventions' restricted and likely transient efficacy in this patient population [28,39,40].

Hemispherectomy is typically regarded as an extreme intervention and is often considered the final resort in managing pharmacoresistant epilepsy, primarily due to its significant functional implications and the potential for complications [41]. Nevertheless, in recent decades, certain reports have indicated that this surgical procedure can be executed with reduced rates of morbidity and mortality [42]. Progress in neurosurgical methodologies has led to advancements in performing smaller resections, facilitating complete functional disconnection with fewer postoperative complications and reduced bleeding.

In addition to the surgical risks, another critical factor influencing decision-making in hemispherectomy surgery is the consideration of its functional consequences. It is recognized that this procedure can lead to permanent deficits, such as hemiparesis, hemianopia, and language disturbances, particularly when performed on the dominant hemisphere. While most deficits may be recoverable with appropriate rehabilitation, preservation of language function is more likely if surgery is conducted early in treatment. Thome et al. thoroughly analyzed 28 neuropsychological assessments with long-term follow-up (mean: 23 months/median: 25.5 months) post-surgery [37]. They found that the preoperative cognitive status remained stable in 18 patients, declined in eight, and improved in two over time. It has been noted previously that most patients maintain their preoperative cognitive status after surgery. The factors predicting favorable cognitive and functional outcomes following surgical treatment in patients with Rasmussen’s encephalitis remain uncertain, lacking consensus in the literature. However, certain reports suggest that early surgery in younger children and patients with an intelligence quotient greater than 70 may be associated with improved motor and cognitive outcomes [40,43].

When there is a concern regarding the preservation of high cortical functions during preoperative evaluation, particularly in older children and cases involving the left hemisphere, the anticipated decline in cognitive functions, despite achieving good seizure control, may deter the decision to proceed with surgery [36,37]. However, it is noteworthy from previous literature that even when hemispherectomy surgery is performed in cases of late-onset disease, the majority of patients maintain their preoperative cognitive status [37]. This finding is particularly intriguing, especially in instances of left-sided hemispherectomy surgery. In Thome et al. study, of 13 patients with hemispherectomy, cognitive status remained unchanged in 9, worsened in 3, and improved in 1, particularly when surgery occurred early, especially in the dominant hemisphere [37]. Patients with poor postoperative functional scores had similar limitations before surgery. Improved hand abilities were mostly observed in patients operated on the left side. Socioeconomic status affected rehabilitation access, potentially hindering cognitive improvement despite active neuroplasticity mechanisms, with only two patients showing cognitive improvement post-surgery [37].

Implications and impending doubts

The introduction of cyclosporine in 1981 significantly improved the success rate of heart transplantation [44,45]. However, this success led to an unexpected problem - a surge in the number of people seeking organ transplantation, which resulted in a relative shortage of available organs [44,45]. Additionally, defining death became increasingly complex. While death was traditionally declared when the heart and lungs stopped functioning, in the 20th century, it became associated with "irreversible coma" as defined by the Ad Hoc Committee of the Harvard Medical School [44,45].

The concept of "brain death" gained prominence, supported by the idea that the human spirit is a product of the brain, not the heart. However, some argue that the identification of brain death was primarily aimed at helping transplant surgeons avoid legal issues [44,45]. Despite medical definitions, instances of people recovering from apparent brain death have been reported, raising questions about the accuracy of determining death solely based on brain activity [44,45].

One documented case involved a woman undergoing surgery for a basilar artery aneurysm. Her body was subjected to hypothermic cardiac arrest, and her heart was stopped temporarily. Objective measurements showed no signs of brain activity. However, after the surgery, her brain activity resumed, and she made a full recovery, challenging traditional notions of irreversible death [3,46].

The increasing number of heart transplants raises questions about potential personality changes in recipients. While such changes have been reported, the topic remains largely unexplored. Questions arise regarding the donor's state of being at the time of organ retrieval and whether the donor's personality traits influence the recipient's acceptance of the transplanted organ.

Further questions delve into cellular memory, including the types of information stored in cells and the potential for a recipient to identify the donor's characteristics encoded in the transplanted organ. These inquiries extend beyond the current understanding of memory and personal identity, suggesting that biological contributions may play a role.

Addressing these questions requires interdisciplinary research beyond death's current medical and legal definitions. Exploring the cellular basis of memory and personal identity could enhance our understanding of heart transplantation and offer insights into broader aspects of neuroscience and identity. This research can potentially improve patient care and integration following organ transplantation.

## Conclusions

Emerging evidence suggests that heart transplantation may involve the transfer of the donor's personality traits and memories to the recipient, challenging conventional views of memory and identity. Additionally, the heart's neural network and bidirectional communication with the brain support the concept of heart-brain connection in memory and personality. Observations from hemispherectomy procedures further highlight the brain's remarkable plasticity. Ethical and philosophical questions regarding the implications of memory transfer in transplantation remain unresolved. Interdisciplinary research is crucial to understanding the complexities of memory transfer, neuroplasticity, and organ integration, offering insights into organ transplantation and broader aspects of neuroscience and human identity.
